# Supplementary material for: The outcomes of patients with kidney failure due to focal segmental glomerulosclerosis (FSGS) in Australia and New Zealand: A cohort study using the Australia and New Zealand Dialysis and Transplant Registry (ANZDATA)
Source: PLoS One. 2023 Nov 2;18(11):e0293721. doi: 10.1371/journal.pone.0293721 (PMC10621846; doi:10.1371/journal.pone.0293721)
Supplement: S1 Table — (DOCX) [file pone.0293721.s001.docx]

| **Primary Renal Disease** | **Frequency** | **Percentage** |
| --- | --- | --- |
| Diabetic nephropathy | 24,258 | 29.5% |
| Autosomal Dominant Polycystic Kidney Disease | 5,223 | 6.4% |
| Analgesic Nephropathy | 3,827 | 4.7% |
| Obstructive Uropathy | 1,408 | 1.7% |
| Lithium Toxicity | 364 | 0.4% |
| Renal Tract Cancer | 588 | 0.7% |
| Renal Stone Disease | 692 | 0.9% |
| Reflux Nephropathy | 3,407 | 4.2% |
| Interstitial Nephritis | 962 | 1.2% |
| Paraproteinaemia | 1,769 | 2.2% |
| Hemolytic Uraemic Syndrome | 305 | 0.4% |
| Hypertensive Nephrosclerosis | 5,852 | 8.2% |
| Renovascular Disease | 3,180 | 3.9% |
| Medullary Cystic Disease | 269 | 0.3% |
| Lead Nephropathy | 136 | 0.2% |
| Cortical Necrosis | 304 | 0.4% |
| Congenital Renal Hypoplasia and Dysplasia | 403 | 0.5% |
| Post-Partum Nephropathy | 364 | 0.4% |
| Sarcoidosis | 43 | 0.1% |
| Gout | 174 | 0.2% |
| Calcineurin inhibitor Toxicity | 221 | 0.3% |
| IgA nephropathy | 4,424 | 5.4% |
| Membranous Nephropathy | 1,051 | 1.3% |
| Primary Focal Segmental Glomerulosclerosis | 2,882 | 3.5% |
| Lupus Nephritis | 870 | 1.1% |
| ANCA Vasculitis | 833 | 1% |
| Henoch-Schonlein Purpura | 136 | 0.2% |
| Membranoproliferative Glomerulonephritis | 1,487 | 1.8% |
| Anti-Glomerular Basement Disease | 579 | 0.7% |
| Presumed /Unclassified Glomerulonephritis | 8,390 | 10.2% |
| Alports | 518 | 0.6% |
| Scleroderma | 216 | 0.3% |
| Uncertain diagnosis | 4,938 | 6% |
| Others | 1,662 | 2.1% |
