# Supplementary material for: The outcomes of patients with kidney failure due to focal segmental glomerulosclerosis (FSGS) in Australia and New Zealand: A cohort study using the Australia and New Zealand Dialysis and Transplant Registry (ANZDATA)
Source: PLoS One. 2023 Nov 2;18(11):e0293721. doi: 10.1371/journal.pone.0293721 (PMC10621846; doi:10.1371/journal.pone.0293721)
Supplement: S2 Table — Abbreviations: FSGS, Focal Segmental Glomerulosclerosis; BMI, body mass index; KRT, kidney replacement therapy. (DOCX) [file pone.0293721.s002.docx]

|  | **Total** | **FSGS** | **Other GD** | **P value** |
| --- | --- | --- | --- | --- |
|  | N=21,386 | N=2,882 | N=18,504 |  |
| **Age (years)** | 50 | 50.4 | 49.9 | 0.215 |
| **Gender** |  |  |  | 0.05 |
| Male | 65% (13,901) | 67% (1,920) | 64% (11,981) |  |
| **Ethnicity** |  |  |  | <0.001 |
| White | 76% (16,430) | 78% (2,240) | 76% (14,190) |  |
| Aboriginal/Torres Strait Islander | 4% (1,009) | 4% (107) | 4% (902) |  |
| Asian | 9% (2,025) | 9% (248) | 9% (1,777) |  |
| Māori | 4% (826) | 4% (101) | 4% (725) |  |
| Pacific | 3% (637) | 4% (115) | 3% (522) |  |
| Other | 2% (369) | 2% (58) | 2% (311) |  |
| Not reported | 0.4% (90) | 0.4% (13) | 0.4% (77) |  |
| **KRT era** |  |  |  | <0.001 |
| 1960-1970 | 2% (400) | 0.3% (8) | 2% (392) |  |
| 1971-1980 | 8% (1,694) | 5% (135) | 8% (1,559) |  |
| 1981-1990 | 14% (2,953) | 12% (339) | 14% (2,614) |  |
| 1991-2000 | 25% (5,320) | 26% (746) | 24% (4,574) |  |
| 2001-2010 | 28% (6,177) | 31% (881) | 28% (5,296) |  |
| 2011-2020 | 22% (4,842) | 27% (773) | 22% (4,069) |  |
| **Smoking status at KRT entry** |  |  |  | 0.469 |
| Current | 14% (2,461) | 13% (344) | 14% (2,117) |  |
| Former | 36% (6,299) | 36% (937) | 36% (5,362) |  |
| Never | 50% (8,634) | 50% (1,265) | 50% (7,369) |  |
| Unknown | 0.2% (29) | 0.1% (2) | 0.2% (27) |  |
| **Diabetes mellitus** | 11% (2,119) | 16% (416) | 10% (1,703) | <0.001 |
| **Chronic lung disease** | 13% (2,360) | 12% (338) | 13% (2,022) | 0.883 |
| **Coronary artery disease** | 22% (4,077) | 25% (644) | 22% (3,433) | 0.002 |
| **Peripheral vascular disease** | 10% (1,829) | 10% (274) | 10% (1,555) | 0.593 |
| **Cerebrovascular disease** | 7% (1,379) | 8% (217) | 7% (1,162) | 0.329 |
| **BMI (kg/m^2^)** | 26.57 | 27.35 | 26.43 | <0.001 |
| **First KRT** |  |  |  | <0.001 |
| Haemodialysis | 70% (14,931) | 67% (1,929) | 70% (13,002) |  |
| Peritoneal dialysis | 30% (6,455) | 33% (953) | 30% (5,502) |  |
| **Follow-up years (standard deviations)** | 9.5 (8.8) | 9.24 (8.36) | 9.54 (8.87) | 0.098 |
| **Native kidney biopsy** | 74% (14,699) | 99% (2,647) | 70% (12,052) | <0.001 |
