# Supplementary material for: The outcomes of patients with kidney failure due to focal segmental glomerulosclerosis (FSGS) in Australia and New Zealand: A cohort study using the Australia and New Zealand Dialysis and Transplant Registry (ANZDATA)
Source: PLoS One. 2023 Nov 2;18(11):e0293721. doi: 10.1371/journal.pone.0293721 (PMC10621846; doi:10.1371/journal.pone.0293721)
Supplement: S3 Table — Abbreviations: FSGS, Focal Segmental Glomerulosclerosis. (DOCX) [file pone.0293721.s003.docx]

| **Cause of death** | **FSGS (%)** | **Non-FSGS (%)** | **Total (%)** |
| --- | --- | --- | --- |
| Cardiovascular | 555 (35.6%) | 19,885 (38.3%) | 20,440 (38.2%) |
| Withdrawal | 298 (19.1%) | 10,571 (20.3%) | 10,869 (20.3%) |
| Cancer | 145 (9.3%) | 3,837 (7.4%) | 3,982 (7.4%) |
| Infection | 218 (14%) | 6,800 (13.1%) | 7,018 (13.1%) |
| Other | 338 (21.7%) | 10,689 (20.6%) | 11,027 (20.6%) |
| Not reported | 6 (0.4%) | 180 (0.4%) | 186 (0.4%) |
