# Supplementary material for: The outcomes of patients with kidney failure due to focal segmental glomerulosclerosis (FSGS) in Australia and New Zealand: A cohort study using the Australia and New Zealand Dialysis and Transplant Registry (ANZDATA)
Source: PLoS One. 2023 Nov 2;18(11):e0293721. doi: 10.1371/journal.pone.0293721 (PMC10621846; doi:10.1371/journal.pone.0293721)
Supplement: S4 Table — Abbreviations: FSGS, Focal Segmental Glomerulosclerosis; GD, Glomerular Diseases; BMI, body mass index; KRT, kidney replacement therapy. (DOCX) [file pone.0293721.s004.docx]

|  | **Total** | **FSGS** | **Other GD** | **P value** |
| --- | --- | --- | --- | --- |
|  | N=11,365 | N=1,469 | N=9,896 |  |
| **Age (years)** | 40.3 | 39.9 | 40.4 | 0.267 |
| **Gender** |  |  |  | 0.760 |
| Male | 66.7% (7,583) | 66.4% (975) | 66.8% (6,608) |  |
| **Ethnicity** |  |  |  | 0.008 |
| Caucasian | 79.6% (9,048) | 81.3% (1,194) | 79.4% (7,854) |  |
| Aboriginal/Torres Strait Islander | 2.7% (304) | 2% (29) | 2.8% (275) |  |
| Asian | 10.9% (1,238) | 9.1% (133) | 11.2% (1,105) |  |
| Māori | 2.5% (282) | 2.2% (32) | 2.5% (250) |  |
| Pacific | 2.4% (270) | 3.3% (49) | 2.2% (221) |  |
| Other | 1.5% (175) | 1.8% (27) | 1.5% (148) |  |
| Not reported | 0.4% (48) | 0.3% (5) | 0.4% (43) |  |
| **KRT era** |  |  |  | <0.001 |
| 1960-1970 | 3.3% (379) | 0.5% (8) | 3.8% (371) |  |
| 1971-1980 | 11.7% (1,328) | 7.9% (116) | 12.3% (1,212) |  |
| 1981-1990 | 16.8% (1,906) | 16.3% (240) | 16.85 (1,666) |  |
| 1991-2000 | 24% (2,731) | 26.3% (387) | 23.7% (2,344) |  |
| 2001-2010 | 27.3% (3,104) | 29.2% (429) | 27% (2,675) |  |
| 2011-2020 | 16.9% (1,917) | 19.7% (289) | 16.5% (1,628) |  |
| **Smoking status at KRT entry** |  |  |  | 0.151 |
| Current | 11.7% (1,026) | 10.1% (126) | 11.9% (900) |  |
| Former | 30.4% (2,674) | 29.5% (370) | 30.5% (2,304) |  |
| Never | 57.9% (5,096) | 60.4% (757) | 57.5% (4,339) |  |
| Unknown | 0.1% (10) | 0.1% (1) | 0.1% (9) |  |
| **Diabetes mellitus** | 4.5% (435) | 6.9% (93) | 4.1% (342) | <0.001 |
| **Chronic lung disease** | 4.5% (435) | 4.2% (56) | 4.6% (379) | 0.807 |
| **Coronary artery disease** | 7.3% (696) | 8.8% (115) | 7.1% (581) | 0.091 |
| **Peripheral vascular disease** | 2.5% (234) | 2.5% (33) | 2.5% (201) | 0.993 |
| **Cerebrovascular disease** | 2.1% (197) | 2% (26) | 2.1% (171) | 0.890 |
| **BMI (kg/m^2^)** | 25.6 | 25.6 | 25.5 | 0.608 |
| **First KRT** |  |  |  | 0.025 |
| Haemodialysis | 65.1% (7,403) | 62% (911) | 65.6% (6,492) |  |
| Peritoneal dialysis | 27.8% (3,164) | 30.6% (449) | 27.4% (2,715) |  |
| Transplant | 7% (798) | 7.4% (109) | 7% (689) |  |
| **Follow-up years (standard deviations)** | 14.4 (9.33) | 14.1 (8.81) | 14.3 (9.40) | 0.484 |
| **Native kidney biopsy** | 81.1% (8,947) | 99.9% (1,416) | 78.3% (7,531) | <0.001 |
