# Supplementary material for: The outcomes of patients with kidney failure due to focal segmental glomerulosclerosis (FSGS) in Australia and New Zealand: A cohort study using the Australia and New Zealand Dialysis and Transplant Registry (ANZDATA)
Source: PLoS One. 2023 Nov 2;18(11):e0293721. doi: 10.1371/journal.pone.0293721 (PMC10621846; doi:10.1371/journal.pone.0293721)
Supplement: S5 Table — Abbreviations: FSGS, Focal Segmental Glomerulosclerosis. * Chronic allograft nephropathy includes chronic antibody and cell mediated rejection, calcineurin inhibitor nephrotoxicity, viral nephritis (such as BK virus), and hypertensive nephrosclerosis. (DOCX) [file pone.0293721.s005.docx]

| **Cause of allograft failure** | **FSGS (%)** | **Non-FSGS (%)** | **Total (%)** |
| --- | --- | --- | --- |
| Acute rejection | 77 (15%) | 1536 (19.8%) | 1613 (19.5%) |
| Chronic allograft nephropathy* | 261 (51%) | 4091 (52.8%) | 4352 (52.6%) |
| Hyperacute rejection | 3 (0.6%) | 108 (1.4%) | 111 (1.3%) |
| Vascular | 27 (5.3%) | 578 (7.4%) | 605 (7.3%) |
| Technical | 13 (2.5%) | 212 (2.7%) | 225 (2.7%) |
| Glomerular disease | 81 (16%) | 366 (4.7%) | 448 (5.4%) |
| Non-adherence | 15 (3%) | 230 (3%) | 245 (3%) |
| Other | 34 (6.6%) | 635 (8.2%) | 669 (8%) |
