# Supplementary material for: The outcomes of patients with kidney failure due to focal segmental glomerulosclerosis (FSGS) in Australia and New Zealand: A cohort study using the Australia and New Zealand Dialysis and Transplant Registry (ANZDATA)
Source: PLoS One. 2023 Nov 2;18(11):e0293721. doi: 10.1371/journal.pone.0293721 (PMC10621846; doi:10.1371/journal.pone.0293721)
Supplement: S6 Table — Abbreviations: FSGS, Focal Segmental Glomerulosclerosis. (DOCX) [file pone.0293721.s006.docx]

| **Cause of death** | **FSGS (%)** | **Non-FSGS (%)** | **Total (%)** |
| --- | --- | --- | --- |
| Cardiovascular | 164 (30.9%) | 4105 (37.8%) | 4269 (37.5%) |
| Withdrawal | 52 (9.8%) | 739 (6.8%) | 791 (6.9%) |
| Cancer | 98 (18.5%) | 1833 (16.9%) | 1931 (17%) |
| Infection | 97 (18.3%) | 1955 (18%) | 2052 (18%) |
| Other | 118 (22.3%) | 2195 (20.2%) | 2313 (20.3%) |
| Not reported | 1 (0.2%) | 27 (0.3%) | 28 (0.3%) |
