# Supplementary figures and images for: The outcomes of patients with kidney failure due to focal segmental glomerulosclerosis (FSGS) in Australia and New Zealand: A cohort study using the Australia and New Zealand Dialysis and Transplant Registry (ANZDATA)
Source: PLoS One. 2023 Nov 2;18(11):e0293721. doi: 10.1371/journal.pone.0293721 (PMC10621846; doi:10.1371/journal.pone.0293721)

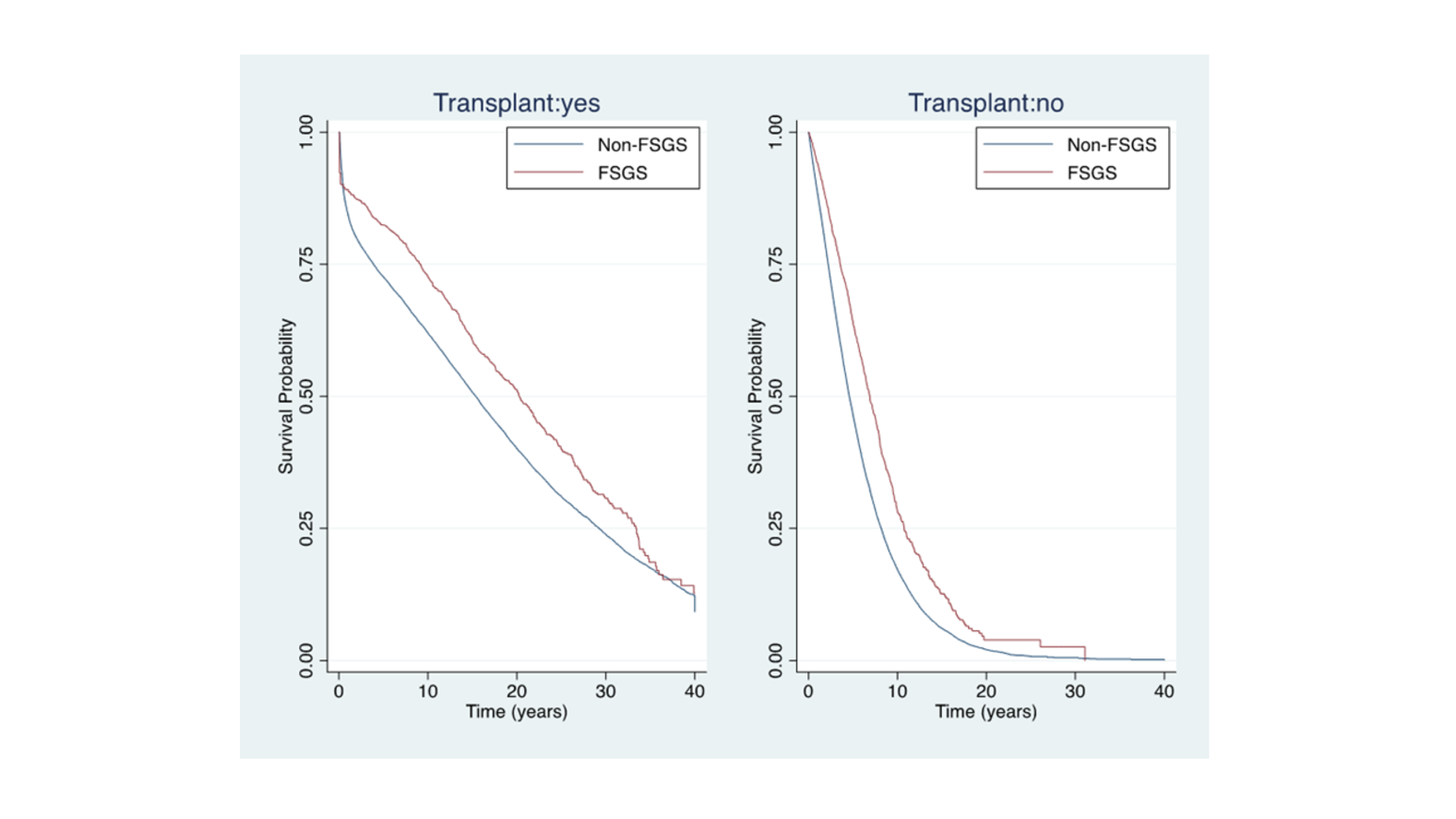

Supplement: S1 Fig — Transplant was considered a time varying covariate. The survival curve between the two groups was not significantly different when kidney transplantation was considered as time-varying covariate (HR 0.99, 95% CI 0.92–1.07, p = 0.856 for no transplant and HR 0.97, 95% CI 0.83–1.14, p = 0.739 for transplant). Abbreviations: FSGS, Focal Segmental Glomerulosclerosis. (TIFF) [file pone.0293721.s007.tiff]

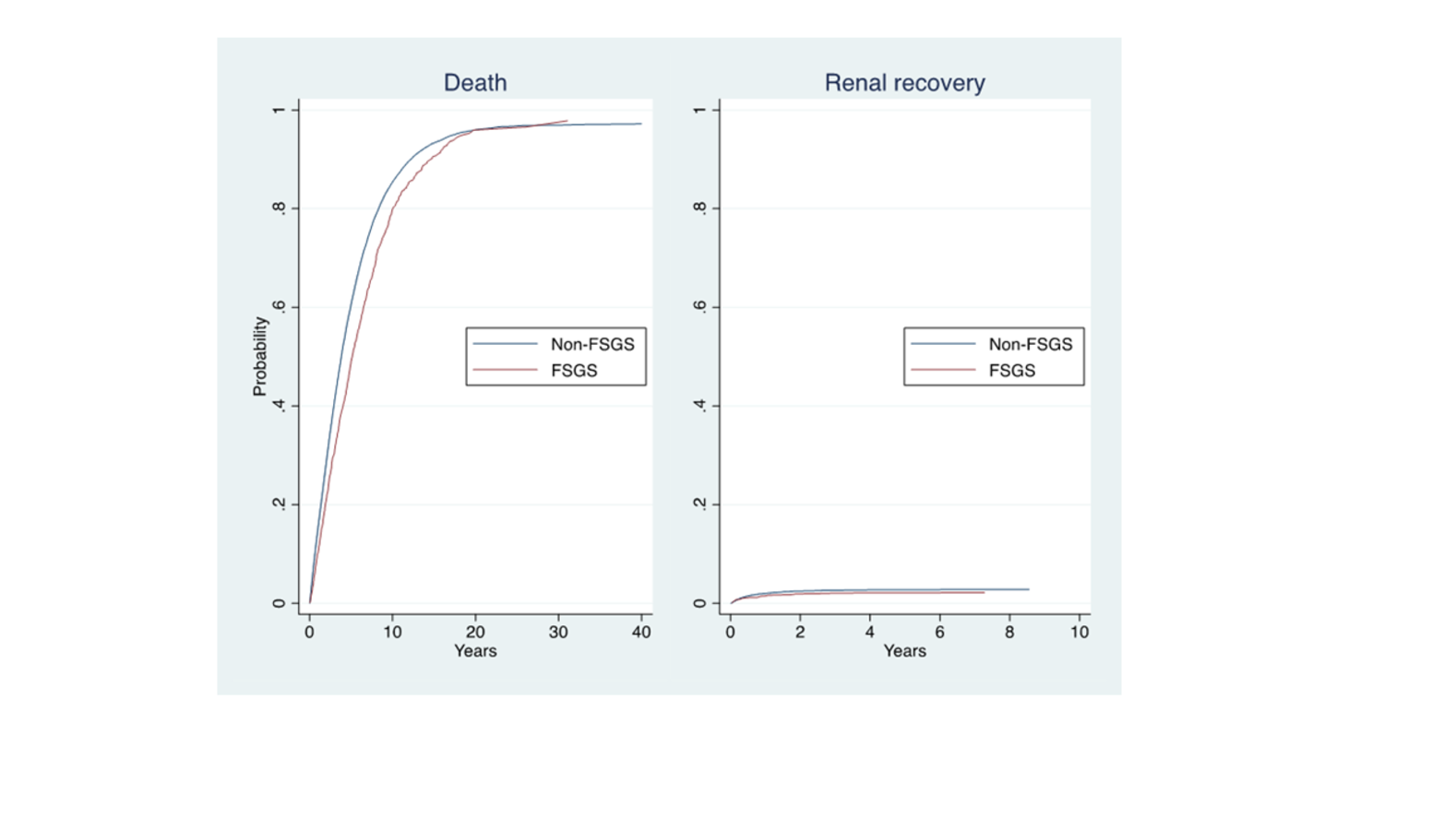

Supplement: S2 Fig — FSGS patients were getting transplant quicker than non-FSGS patients. The difference between the two groups was significant p < 0.001. Abbreviations: FSGS, Focal Segmental Glomerulosclerosis. (TIFF) [file pone.0293721.s008.tiff]

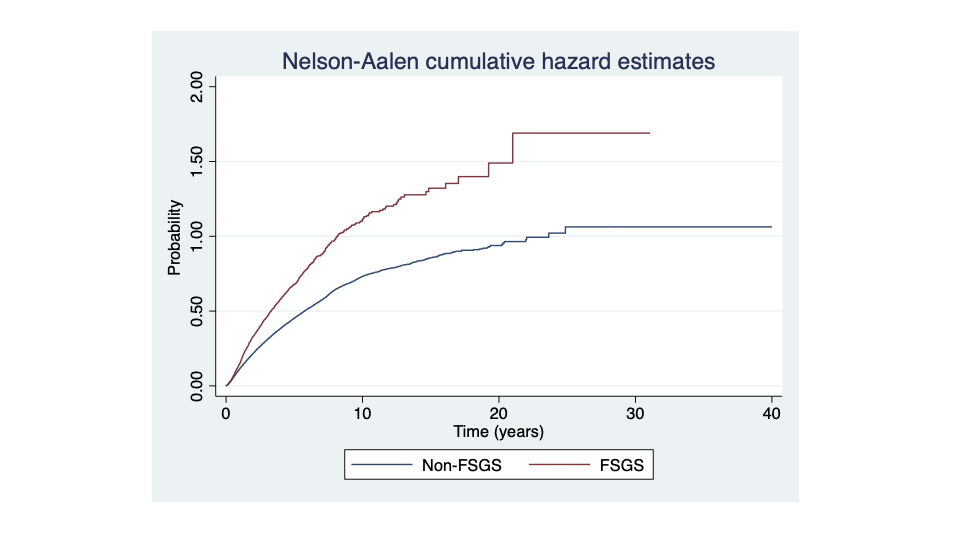

Supplement: S3 Fig — The cumulative incidence of death was higher for non-FSGS than FSGS, while the cumulative incidence of kidney transplantation was appreciably higher in the FSGS group, that is non-FSGS patients were more likely to die before receiving a transplant (SHR 1.51, 95% CI 1.43–1.59, p<0.001). Abbreviations: FSGS, Focal Segmental Glomerulosclerosis. (TIFF) [file pone.0293721.s009.tiff]

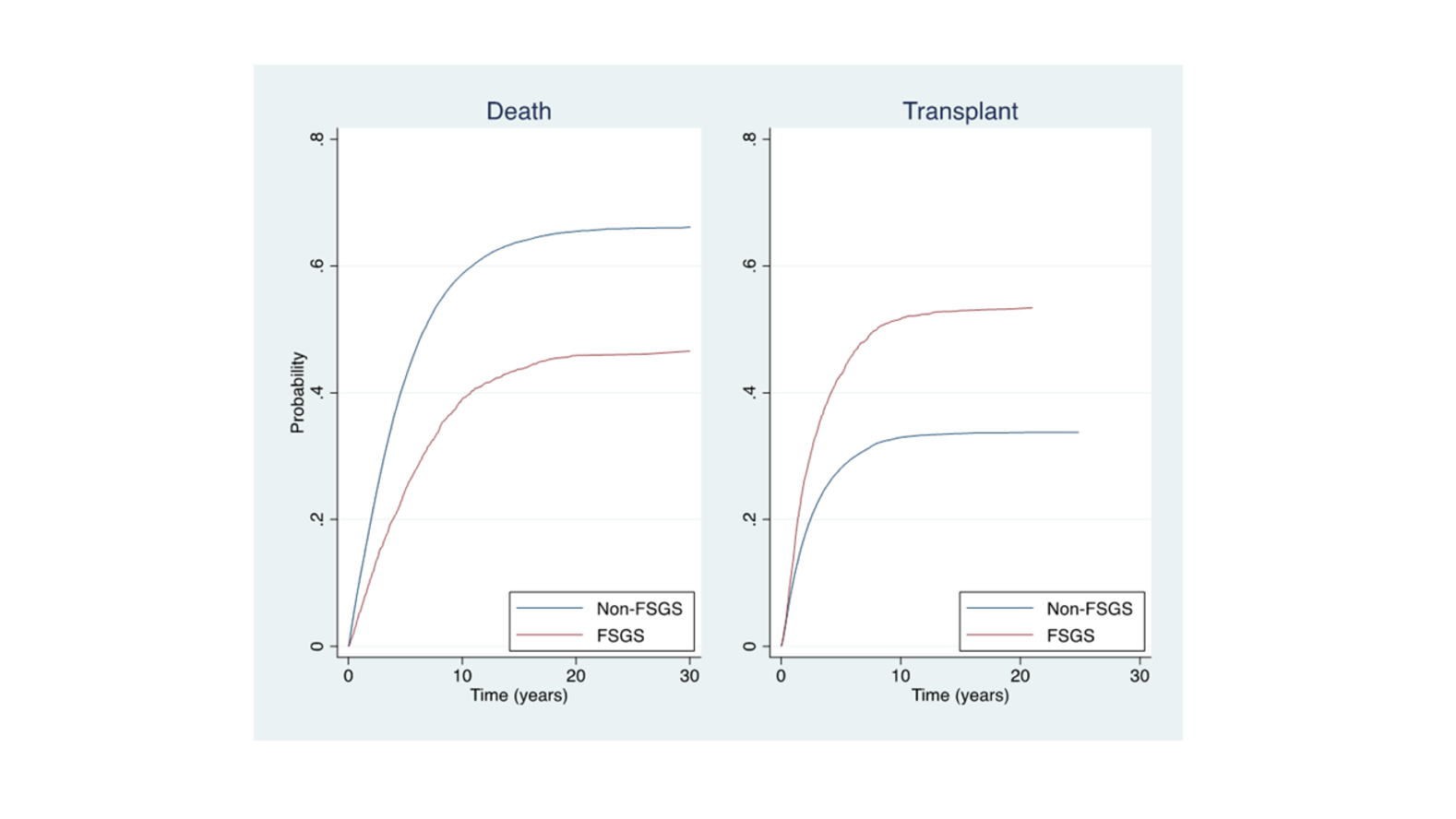

Supplement: S4 Fig — First kidney allograft survival was similar between both groups when death was considered as competing risk (SHR 1.09, 95% CI 0.94–1.28, p = 0.26). Abbreviations: FSGS, Focal segmental glomerulosclerosis. (TIFF) [file pone.0293721.s010.tiff]

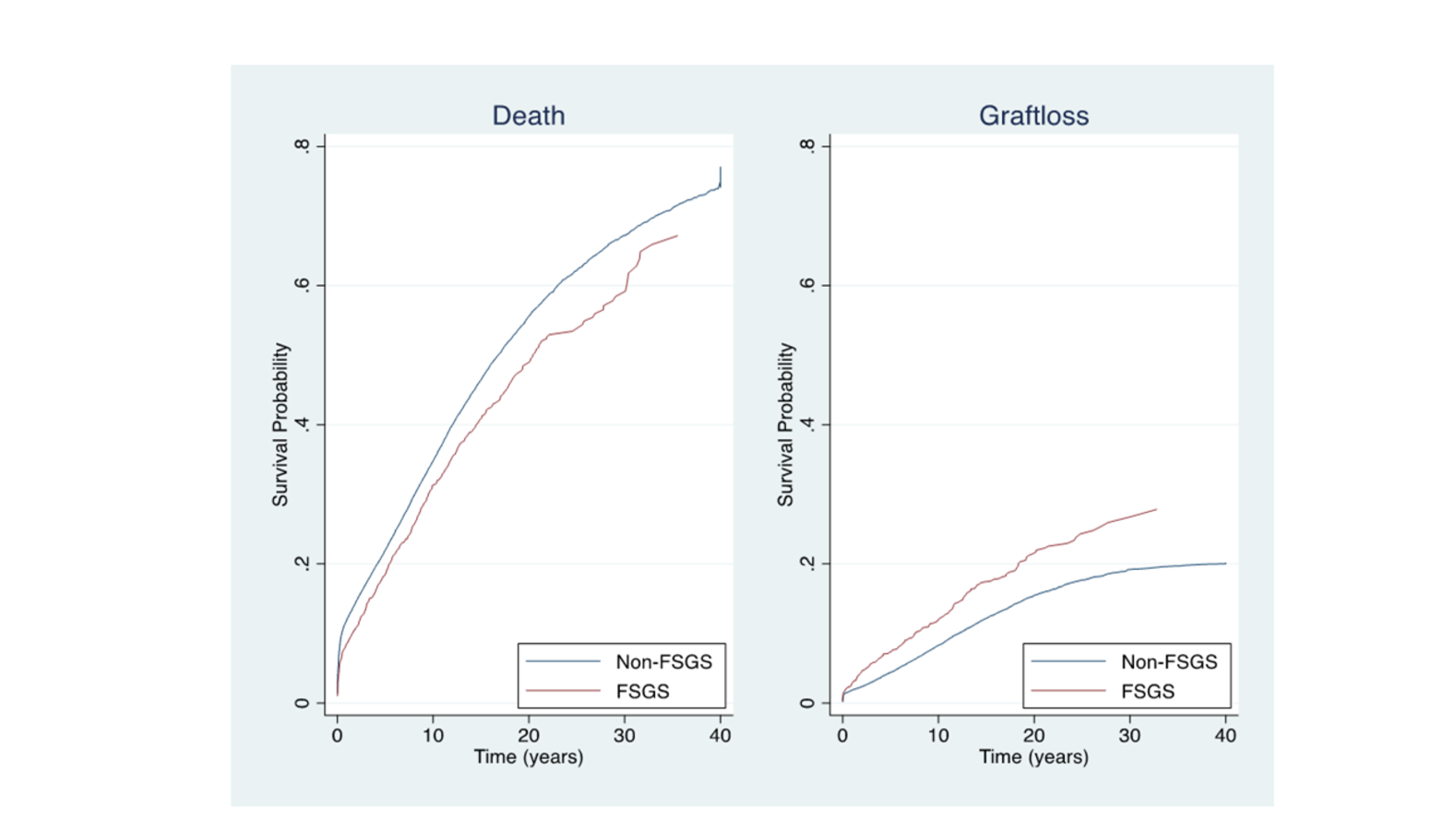

Supplement: S5 Fig — First kidney allograft survival was similar between both groups when adjusted for disease recurrence (with no recurrence SHR 1.09, 95% 0.93–1.27, p = 0.27 and with recurrence SHR 1.28, 95% CI 0.96–1.69, p = 0.09). Abbreviations: FSGS, Focal segmental glomerulosclerosis. (TIFF) [file pone.0293721.s011.tiff]

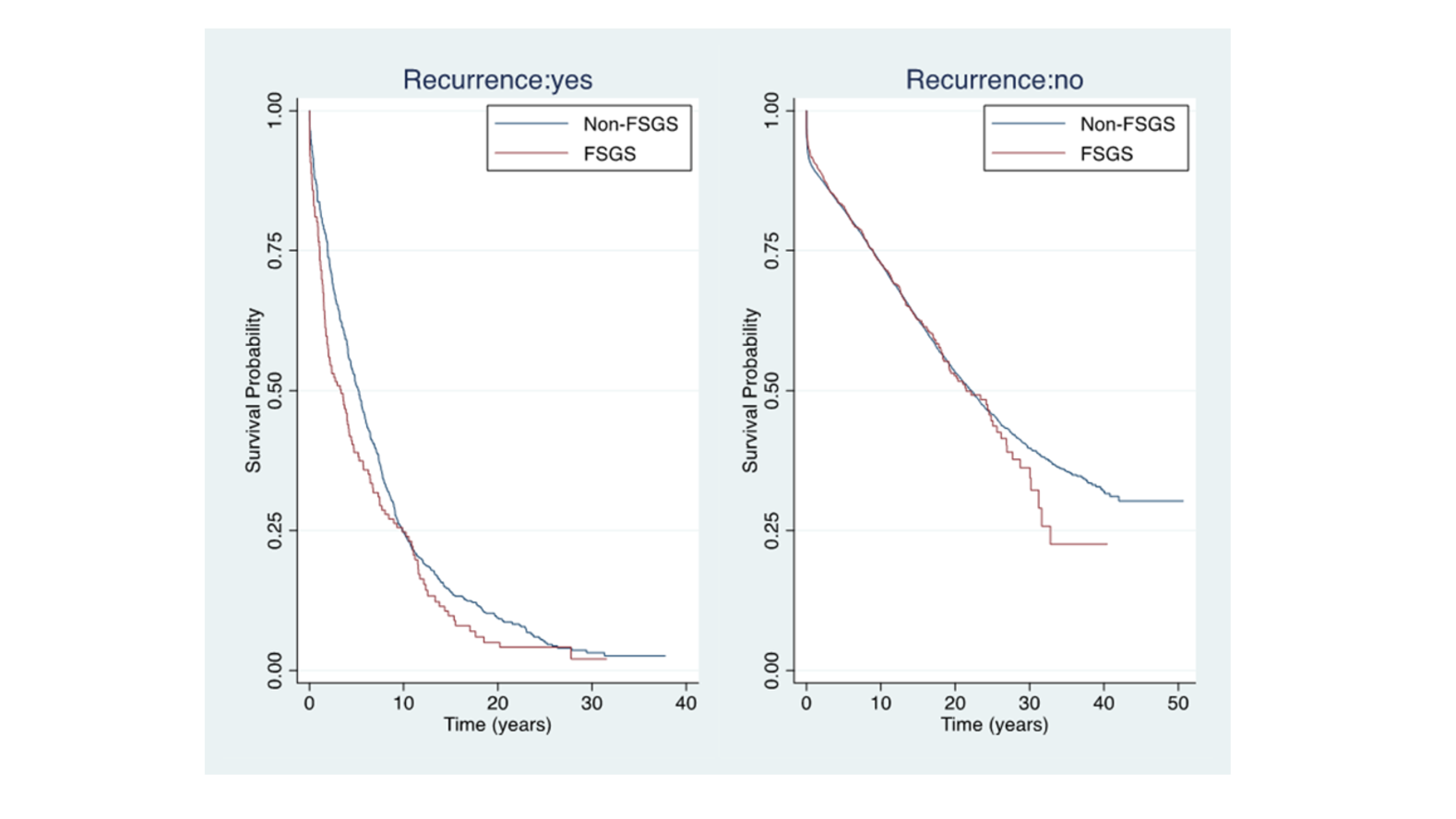

Supplement: S6 Fig — There was no significant difference between the two groups when graft loss was considered as a time-varying risk modifier (HR 0.95, 95% CI 0.76–1.19, p = 0.66 for no graft loss and HR 1.22, 95% CI 0.98–1.52, p = 0.07 for graft loss). Abbreviations: FSGS, Focal segmental glomerulosclerosis. (TIFF) [file pone.0293721.s012.tiff]

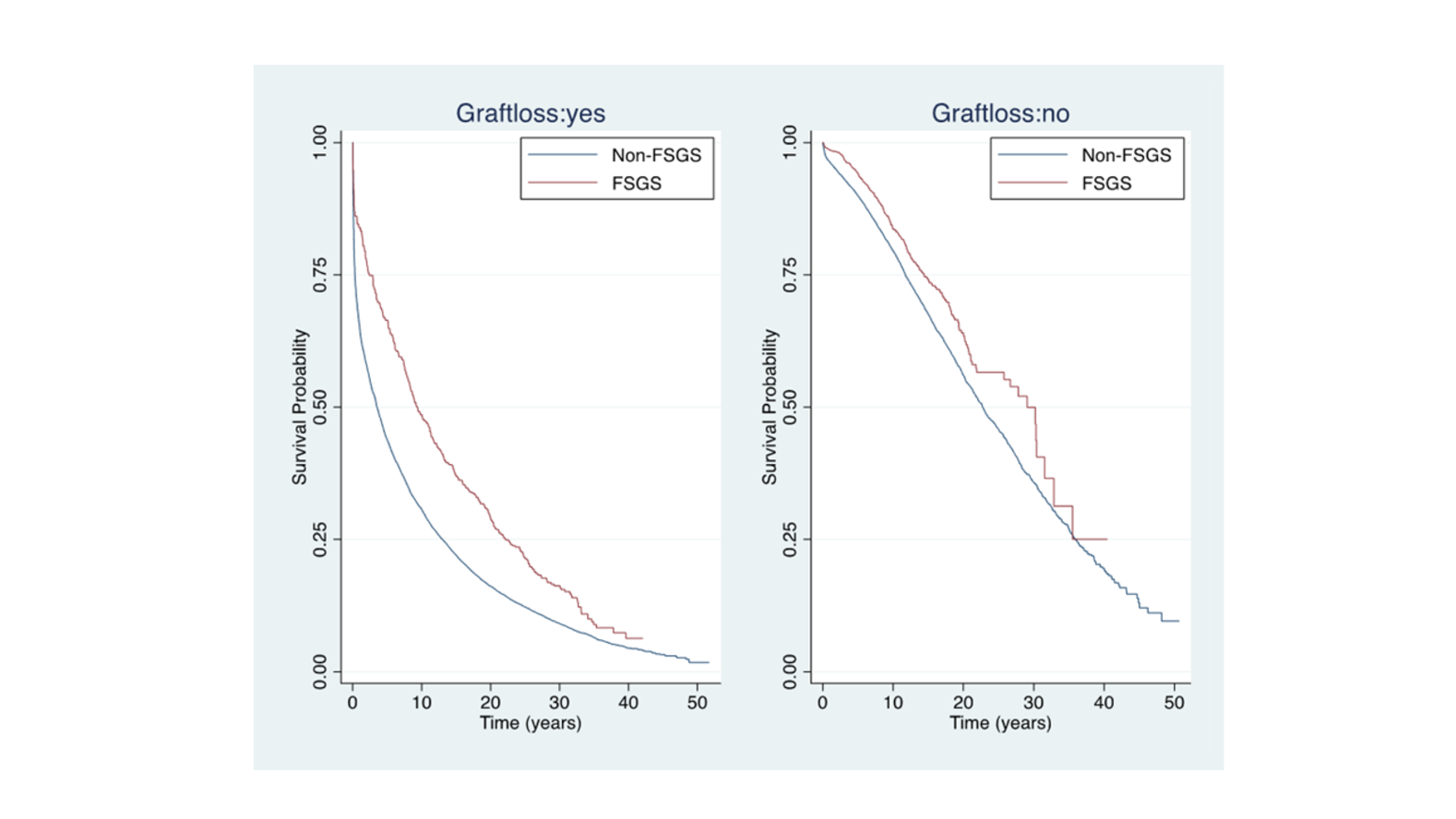

Supplement: S7 Fig — (TIFF) [file pone.0293721.s013.tiff]
