# Supplementary material for: The outcomes of patients with kidney failure due to focal segmental glomerulosclerosis (FSGS) in Australia and New Zealand: A cohort study using the Australia and New Zealand Dialysis and Transplant Registry (ANZDATA)
Source: PLoS One. 2023 Nov 2;18(11):e0293721. doi: 10.1371/journal.pone.0293721 (PMC10621846; doi:10.1371/journal.pone.0293721)
Supplement: Attachment — Submitted filename: PONE-D_22-23253R.pdf [file pone.0293721.s018.pdf]

The outcomes of patients with kidney failure due to focal segmental glomerulosclerosis (FSGS) in Australia and New Zealand: A cohort study using the Australia and New Zealand Dialysis and Transplant Registry (ANZDATA)

Manuscript Number: **PONE-D-22-23253R2**

Comments to Authors and Editor

The authors attempt to investigate the characteristics, treatments, and outcomes of all cases of kidney failure due to FSGS in the Australian and New Zealand populations, using 2018 data from the Australia and New Zealand Dialysis and Transplant (ANZDATA) Registry. Survivals between FSGS vs non-FSGS look very promising. However, I have concerns regarding the clarity of the presented data, as readers may struggle to replicate the described approach in other datasets. Therefore, I believe that the paper would benefit from revisions to the methodology and outcomes to enhance the understanding of the differences in survival rates between the two disease groups.

Specific comments

Number of participants: The FSGS and non-FSGS groups have a significant difference in the number of participants (with n=2882 and n=79,752, respectively). This can result in biased estimates of survival probabilities and hazard ratios, which may lead to incorrect conclusions about the relationship between the exposure and survival outcomes.

Outcomes: Outcome 1: The authors have already defined the kidney transplantation as a censor event in line 108, but not sure why transplantation was considered as censored event in line 109. I am not sure about the meaning of statement "Transplantation was considered a censoring event or time-varying covariate", especially the time varying covariate. Outcome 2: What is the reason for investigating the second outcome, which is "dialysis-independent recovery of kidney function"? the number of cases for this condition is minimal, I believe. Outcome 3: I am also uncertain about the inclusion of kidney transplantation as a third outcome in the study. It appears to diverge from the main aim and objectives of the research, which focus on the impact of FSGS on outcomes such as death and graft failure. Outcome 4: The ANZDATA Registry records the primary renal disease at the start of the study. As such, I am uncertain how the recurrence of the primary disease was analysed using ANZDATA. With regards to Outcome 5, I am unsure of the rationale behind using the commencement of dialysis as an endpoint. Typically, the standard measure for graft survival is from the first kidney transplant until the failure of the graft. The authors noted that death was treated as a competing event, and it is unclear why death was censored for outcomes 2-5.

Statistical analysis: The statistical analysis plan used in the study lacks clarity, as the authors have mixed up standard survival models with competing risk analysis. Additionally, there is a lack of detailed information on the use of time-varying covariates in the survival analysis.

Results: The manuscript contains numerous findings, and the authors have included irrelevant outcomes, resulting in an unnecessarily lengthy document.

Timeframe: The ANZDATA 2018 data appears to be quite outdated. It is recommended that the authors use more recent ANZDATA 2022 data to obtain a more accurate depiction of the latest findings. This would enable them to present the actual outcomes of the recent data more effectively.
