# Supplementary material for: The outcomes of patients with kidney failure due to focal segmental glomerulosclerosis (FSGS) in Australia and New Zealand: A cohort study using the Australia and New Zealand Dialysis and Transplant Registry (ANZDATA)
Source: PLoS One. 2023 Nov 2;18(11):e0293721. doi: 10.1371/journal.pone.0293721 (PMC10621846; doi:10.1371/journal.pone.0293721)
Supplement: Attachment — Submitted filename: Rebuttal Letter.docx [file pone.0293721.s016.docx]

17/10/2022

Dr Bhadran Bose

Department of Nephrology,

Nepean Hospital

Kingswood NSW 2747,

Australia

Email: [bhadran.bose@health.nsw.gov.au](mailto:bhadran.bose@health.nsw.gov.au)

Dr Serena Maria Bagnasco

Academic Editor

PLOS ONE

Dear Dr Bagnasco

Re: Resubmission of the manuscript titled, **“The outcomes of patients with kidney failure due to focal segmental glomerulosclerosis (FSGS) in Australia and New Zealand: A cohort study using the Australia and New Zealand Dialysis and Transplant Registry (ANZDATA)” – PONE-D-22-23253**

We thank the reviewers and editor for their valuable feedback. We have addressed the comments raised by the editor and reviewers in this letter. In addition, we have highlighted our response to each of the comments.

***Additional Editor Comments:***

Comment 1: The manuscript has significant flaws and is not acceptable in its current form.
The study is based on data extracted from the ANZDATA Registry which was collected over a period of more than 50 years, during which, many aspects of diagnostic and clinical approach to kidney transplantation have changed, which may result in too broad heterogeneity of information for appropriate in-depth analysis.

**Response: We agree, that many aspects of dialysis and transplantation have changed over the last 50 years, which may have confounded the findings. In order to account for this, we have adjusted for dialysis or transplant era in our analysis.**

Comment 2: Important data are missing for example the number of patients for each major cause of ESRD (Hypertension, Diabetes, congenital uropathies, polycystic kidney …), many of these diseases do not recur.

**Response: We have added a table (S1 Table) on pages 1 and 2 of the supplementary material giving a break down of the causes of kidney failure in patients whose first kidney replacement therapy was dialysis in Australia and New Zealand.**

Comment 3: Only 25% of the patients with “GN” had a native biopsy, the number of patients for specific type of glomerular diseases is not provided and, absent this information, analysis of the rate of recurrence between FSGS and non-FSGS or GN group is meaningless.

**Response: As per S3 Table, 99.9% of FSGS patients and 78.3% of patients with other forms of glomerular disease had a native kidney biopsy.**

**We agree that analysis of the rates of post-transplant recurrence between FSGS and non-FSGS is fraught. Hence, we have deleted that paragraph.**

**However, we feel that comparison between FSGS and other glomerular disease is useful. As such, we have added the following paragraph (page 16, line 296 to 299).**

**“*With respect to post-transplant recurrence rates, we only compared the recurrence rates between patients with FSGS and those with kidney failure due to other GD. We did not compare FSGS versus non-FSGS as some of the non-FSGS causes of kidney failure do not recur post-transplant.*”**

Comment 4: Although the majority of FSGS patients had a biopsy in the native disease, it is not clear how recurrence was diagnosed.

**Response: Recurrence was diagnosed based on the treating nephrologists’ reports to ANZDATA. However, the frequency of FSGS recurrence in kidney allografts may have been under-estimated as not all patients with FSGS who experienced kidney allograft failure received a kidney biopsy.**

**This has been stated as one of the limitations in our discussion (page 22, line 469 to 471).**

**“*The frequency of FSGS recurrence in kidney allografts may have been under-estimated as not all patients with FSGS who experienced kidney allograft failure received a kidney biopsy*.”**

Comment 5: Most of the tables need to be streamlined into a more concise format, the current one makes it impossible for the reader to review the difference, and its significance, among the main category of data presented.

**Response: We have streamlined the tables (Table 1, Table 2, S2 Table, S4 Table) by deleting age categories, BMI categories and number of female patients.**

Comment 6: Figures need titles, and the level of significance is often lacking in the legend.

**Response: Titles had already been provided for all figures (Fig 1-3, S1-S7 Fig). We have added hazard ratio with 95% confidence interval and p value to each figure.**

***Reviewers' comments:***

***Reviewer #1:*** 

Major concerns:

Comment 1: It is not clear why the authors chose to compare patients with FSGS with all other patients with any cause of ESKD. The authors did not clarify the purpose of this work.

**Response: We chose to compare patients with FSGS with all other patients with any cause of kidney failure because this reflects a common question asked by patients (i.e. “what is the prognosis for patients with my condition compared to other patients starting dialysis or having a kidney transplant?”). Nonetheless, we acknowledge the Reviewer’s implied concern regarding the heterogeneity of all other patients with any cause of kidney failure. To address this, we have also performed a subgroup analysis comparing patients with FSGS those with other forms of glomerular disease, as this more closely approximates a “like with like” comparison. This has been mentioned in the methods section in page 5, line 105 to 108.**

Comment 2: The authors showed that only 25% of the non-FSGS group had kidney biopsy. Considering the large number of patients in this group, it is possible that some of these patients have FSGS as well.

**Response: We acknowledge that this is possible and have specifically stated this as one of the limitations in our discussion (page 22, line 472-473).**

**“As the patients’ nephrologists determined the diagnosis of FSGS and comorbidities, coding bias is possible”**

Comment 3: Considering that FSGS can be a secondary disease, it is possible that the FSGS group may have had secondary FSGS, that is due another kidney injury and not primary FSGS. It is also not clear if the authors aim to study the primary FSGS only or any type of FSGS.

**Response: We have already acknowledged the possibility of coding (misclassification) bias in the limitation section of the Discussion (page 22, line 471 to 473).**

**“Like other registries, ANZDATA is a voluntary registry, and there is no external audit of data accuracy. As the patients’ nephrologists determined the diagnosis of FSGS and comorbidities, coding bias is possible”**

Comment 4: The authors showed most of the parameters have statistically significant P value, however, reviewing the numbers it does not seem to be the case.

**Response: We have confirmed that all calculated p values are correct.**

Comment 5: Most of published data showed that the allograft survival is less in patients with FSGS due to high risk of recurrence, this study showed when death was analyzed as a competing risk, the survival in both groups was similar. It is not clear why the death is a factor in allograft survival in this group who has very high rate of recurrent disease and allograft failure due to recurrence.

**Response: Death was considered as a competing risk because death precludes observation of the event of interest, that is, if a patient dies before a graft failure, we will not be able to observe graft loss. On the other hand, we acknowledge that the differing rate of recurrence should be considered; indeed 11% of FSGS patients experienced recurrence compared to 2.5% of non- FSGS patients. We have added the results of analysis adjusting for recurrence as an effect modifier and have reported results separately for patients who experienced recurrence and those who did not (page 17, line 333 to 336).**

**“*Similar results were noted when recurrence of primary disease was considered as an effect modifier (with no recurrence SHR 1.09, 95% 0.93-1.27, p=0.27 and with recurrence SHR 1.28, 95% CI 0.96-1.69, p=0.09).*”**

Comment 6: The authors showed that recurrent disease is higher in the FSGS group comparing to non-FSGS group, 11% vs 2.5 %. The recurrence rate of FSGS appears much lower than any published data, which raises the concern if really all these cases were primary FSGS. Additionally, what diseases are considered to be recurred post-transplant in the non-FSGS group?

Considering that these findings are significantly different from well-established data, the authors did not show any explanation to their results.

**Response: We respectfully disagree with the reviewer. In the literature, the reported rates of recurrent FSGS in adult population are wide. In registry-based studies, the recurrence rate is between 9% to 15%(1-3) and in smaller studies it is between 17% to 55%(4-7). This is similar to what we have observed in our registry-based study.**

Comment 7: The findings of this work are not clinically relevant and do not add much to our understanding of FSGS as compared to other causes of ESKD.

**Response: We respectfully disagree. It is the first time most of the outcomes are being compared between FSGS and non-FSGS in the Australian/New Zealand population. The results of this study will help better inform discussions and shared decision-making between clinicians, patients with FSGS initiating KRT, and their caregivers.**

Minor concerns:

Having the tables inside the text makes the manuscript harder to read.

**Response: This was done as per the journal requirements.**

***Reviewer #2:*** 

I only have minor observations:

Comment 1: Table 1 (and tables in general): please use standard approach to decimals in percentages

**Response: We have made relevant changes where appropriate. Thanks**

Comment 2: Page 18: It's actually meaningless to compare recurrence rate of FSGS with non-FSGS, as the latter group includes diseases that can't recur after tx (i.e., ADPK). in addition to this analysis, the Authors correctly show that FSGS had higher recurrence rate compared to other forms of GN (supplementary materials): I would only include this analysis which is much more meaningful

**Response: We have deleted the paragraph comparing FSGS versus non-FSGS but included the following sentence to explain the reasoning behind the exclusion main manuscript (page 16, line 296 to 299).**

**“*****With respect to post-transplant recurrence rates, we only compared the recurrence rates between patients with FSGS and those with kidney failure due to other GD. We did not compare FSGS versus non-FSGS as some of the non-FSGS causes of kidney failure do not recur post-transplant*.”**

**We have also deleted S5-S6 Fig.**

Comment 3: Supplementary table 2: please provide p values: in the text (page 11) is stated that no difference are observed in the causes of death, but some of these numbers seems actually different between groups

**Response: A p value of 0.02 was obtained from a Chi-square test and it shows there was statistically significant different between the two groups. We have changed our statement as below. We thank the reviewer for pointing this out. This has been added in page 10, line 196-197.**

**“*There were more cardiovascular related deaths in the non-FSGS patients and more cancer related deaths in the FSGS patients (p=0.02)”***

Comment 4: Supplementary figure 3: please double check the figure: it seems that xy axis are not correct

**Response: We agree with the reviewer that graph reads wrongly as y axis looks inverted, hence we have deleted the Kaplan Meir plot and instead added a Nelson-Aalen cumulative hazard estimates plot which shows that the probability of undergoing transplant increases with time and then plateaus. We thank the reviewer for pointing this one out.**

Comment 5: Supplementary table 4: correct typo in line 2 column 4

**Response: Typo corrected. Thanks**

Comment 6: Supplementary table 4: Chronic allograft nephropathy is an obsolete definition (eliminated from Banff about a decade ago). I understand that grouping different diagnosis from such different time periods can be hard, but I would suggest to explain that CAN actually includes different etiologies that have been better defined over time (chronic ab and cell mediated rejection, CNI toxicity, viral nephritis, etc)

**Response: We have added a sentence below the table explaining what chronic allograft nephropathy means in Page 9 of the supplementary material.**

**“Chronic allograft nephropathy includes chronic antibody and cell mediated rejection, calcineurin inhibitor nephrotoxicity, viral nephritis (such as BK virus), and hypertensive nephrosclerosis”**

Along with this letter, we are resubmitting the main manuscript and the supplementary material (both clean versions and with track changes).

Once again, we thank you for reviewing our manuscript. We look forward to hearing the outcome of your editorial review in the near future.

Sincerely

Dr Bhadran Bose

On behalf of the authors

1.	Allen PJ, Chadban SJ, Craig JC, Lim WH, Allen RDM, Clayton PA, et al. Recurrent glomerulonephritis after kidney transplantation: risk factors and allograft outcomes. Kidney Int. 2017;92(2):461-9.

2.	Francis A, Trnka P, McTaggart SJ. Long-Term Outcome of Kidney Transplantation in Recipients with Focal Segmental Glomerulosclerosis. Clin J Am Soc Nephrol. 2016;11(11):2041-6.

3.	Jiang SH, Kennard AL, Walters GD. Recurrent glomerulonephritis following renal transplantation and impact on graft survival. BMC Nephrol. 2018;19(1):344.

4.	Senggutuvan P, Cameron JS, Hartley RB, Rigden S, Chantler C, Haycock G, et al. Recurrence of focal segmental glomerulosclerosis in transplanted kidneys: analysis of incidence and risk factors in 59 allografts. Pediatr Nephrol. 1990;4(1):21-8.

5.	Banfi G, Colturi C, Montagnino G, Ponticelli C. The recurrence of focal segmental glomerulosclerosis in kidney transplant patients treated with cyclosporine. Transplantation. 1990;50(4):594-6.

6.	Ingulli E, Tejani A. Incidence, treatment, and outcome of recurrent focal segmental glomerulosclerosis posttransplantation in 42 allografts in children--a single-center experience. Transplantation. 1991;51(2):401-5.

7.	Dantal J, Baatard R, Hourmant M, Cantarovich D, Buzelin F, Soulillou JP. Recurrent nephrotic syndrome following renal transplantation in patients with focal glomerulosclerosis. A one-center study of plasma exchange effects. Transplantation. 1991;52(5):827-31.
