# Supplementary material for: The outcomes of patients with kidney failure due to focal segmental glomerulosclerosis (FSGS) in Australia and New Zealand: A cohort study using the Australia and New Zealand Dialysis and Transplant Registry (ANZDATA)
Source: PLoS One. 2023 Nov 2;18(11):e0293721. doi: 10.1371/journal.pone.0293721 (PMC10621846; doi:10.1371/journal.pone.0293721)
Supplement: Attachment — Submitted filename: Rebuttal Letter 23 11 2022.docx [file pone.0293721.s017.docx]

23/11/2022

Dr Bhadran Bose

Department of Nephrology,

Nepean Hospital

Kingswood NSW 2747,

Australia

Email: [bhadran.bose@health.nsw.gov.au](mailto:bhadran.bose@health.nsw.gov.au)

Dr Rajendra Bhimma

Academic Editor

PLOS ONE

Dear Dr Bhimma

Re: Resubmission of the manuscript titled, **“The outcomes of patients with kidney failure due to focal segmental glomerulosclerosis (FSGS) in Australia and New Zealand: A cohort study using the Australia and New Zealand Dialysis and Transplant Registry (ANZDATA)” – PONE-D-22-23253**

We thank the reviewers and editor for their valuable feedback. We have addressed the comments raised by the reviewers in this letter. In addition, we have highlighted our response to each of the comments.

Along with this letter, we are resubmitting the main manuscript (both clean versions and with track changes).

Reviewer #2: The Authors included several improvements to the the manuscript. However, I still have few observations

1 Table 1: Percentage of native kidney biopsy: doesn't make much sense to compare FSGS with non-FSGS, as the latter will include a relevant proportion of diagnosis for which a renal biopsy is never performed (i.e., ADPKD); this data is meaningful only in STable 2
**Response: We thank the reviewer for the valuable comment. We have deleted the row containing percentage of native kidney biopsy in Table 1.**

- Line 186: comment on significance of the data

**Response: We noted more cardiovascular deaths in non-FSGS patients, which could be since the non-FSGS group were older patients with more prevalence of diabetes and coronary artery disease.**

**FSGS group had more cancer-related deaths, which could be due to long term immunosuppression exposure but hard to comment as we don't have information regarding their treatment before commencing kidney replacement therapy.**

- Line 221: is this paragraph relevant? numbers seem to be fairly low here
**Response: We thank the reviewer for their comment, and we have deleted this paragraph**

- Line 243: recurrence paragraph in results need major attention as no data on recurrence are actually explained here. Please amend.

**Response: We thank the reviewer for highlighting this point. We have amended the manuscript describing the recurrence rates between FSGS and other GD. Line 266-268**

***“FSGS was associated with an increased risk of recurrence of primary kidney disease when compared to other forms of GD (aHR 1.67, 95% CI 1.30-2.14, p<0.001). Competing risk analysis showed similar results (SHR 1.30, 95% CI 1.05-1.60, p=0.02).”***

- Line 322: these data were not shown anywhere in the text

**Response: Based on the initial reviewer’s comment (from first revision) we had deleted the analysis comparing recurrence between FSGS and non-FSGS patients as it was felt to be meaningless by the reviewer. We should have deleted the sentence describing that analysis in our discussion. However, we have now changed the sentence. Line 320-322**

***“Following first kidney transplantation, allograft survival and patient survival were also comparable between FSGS and non-FSGS patients.”***

Once again, we thank you for reviewing our manuscript. We look forward to hearing the outcome of your editorial review in the near future.

Sincerely

Dr Bhadran Bose

On behalf of the authors
