# Supplementary material for: The outcomes of patients with kidney failure due to focal segmental glomerulosclerosis (FSGS) in Australia and New Zealand: A cohort study using the Australia and New Zealand Dialysis and Transplant Registry (ANZDATA)
Source: PLoS One. 2023 Nov 2;18(11):e0293721. doi: 10.1371/journal.pone.0293721 (PMC10621846; doi:10.1371/journal.pone.0293721)
Supplement: Attachment — Submitted filename: Rebuttal Letter PLOSONE 06 06 2023.docx [file pone.0293721.s019.docx]

06/06/2023

Dr Bhadran Bose

Department of Nephrology,

Nepean Hospital

Kingswood NSW 2747,

Australia

Email: [bhadran.bose@health.nsw.gov.au](mailto:bhadran.bose@health.nsw.gov.au)

Dr Rajendra Bhimma

Academic Editor

PLOS ONE

Dear Dr Bhimma

Re: Resubmission of the manuscript titled, **“The outcomes of patients with kidney failure due to focal segmental glomerulosclerosis (FSGS) in Australia and New Zealand: A cohort study using the Australia and New Zealand Dialysis and Transplant Registry (ANZDATA)” – PONE-D-22-23253**

We thank the reviewers for their comments and feedback. We had initially submitted our manuscript to PLOS ONE on August 19, 2022. Since then, we have had multiple revisions based on the comments and feedback from various reviewers. Following our latest submission, reviewer 5 has given some new comments. In this resubmission we will try to address most of the comments raised by reviewer 5.

Reviewer #4: All comments previously made have been satisfactorily answered.
However, the authors may wish to correct "Pacific" to "Pacific Islander" in the Ethnicity category of all relevant patients characteristics tables.

**RESPONSE: We thank the reviewer for this comment. We have changed the term “Pacific” to Pacific Islander” in the revised version of the manuscript.**

Reviewer #5:  The authors attempt to investigate the characteristics, treatments, and outcomes of all cases of kidney failure due to FSGS in the Australian and New Zealand populations, using 2018 data from the Australia and New Zealand Dialysis and Transplant (ANZDATA) Registry.

Survivals between FSGS vs non-FSGS look very promising. However, I have concerns regarding the clarity of the presented data, as readers may struggle to replicate the described approach in other datasets. Therefore, I believe that the paper would benefit from revisions to the methodology and outcomes to enhance the understanding of the differences in survival rates between the two disease groups.

**RESPONSE: We thank the reviewer for this comment. We are unsure which aspect of the methodology and outcomes the reviewer would like us to revise. This manuscript's methodology and outcomes are similar to the previously published ANZDATA study.**

***Yang WL, Bose B, Zhang L, McStea M, Cho Y, Fahim M, et al. Long-term outcomes of patients with end-stage kidney disease due to membranous nephropathy: A cohort study using the Australia and New Zealand Dialysis and Transplant Registry. PLoS One. 2019;14(8):e0221531***

***Zhang L, Liu X, Pascoe EM, Badve SV, Boudville NC, Clayton PA, et al. Long-term outcomes of end-stage kidney disease for patients with IgA nephropathy: A multi-centre registry study. Nephrology (Carlton). 2016;21(5):387-96.***

***Tang W, Bose B, McDonald SP, Hawley CM, Badve SV, Boudville N, Brown FG, Clayton PA, Campbell SB, Peh CA, Johnson DW. “The outcomes of patients with end-stage renal disease and ANCA-associated vasculitis in Australia and New Zealand.” Clinical Journal of the American Society of Nephrology. Clin J Am Soc Nephrol. 2013 May;8(5):773-80. doi: 10.2215/CJN.08770812. Epub 2013 Jan 24***

Specific comments

Number of participants: The FSGS and non-FSGS groups have a significant difference in the number of participants (with n=2882 and n=79,752, respectively). This can result in biased estimates of survival probabilities and hazard ratios, which may lead to incorrect conclusions about the relationship between the exposure and survival outcomes.

**RESPONSE: We thank the reviewer for this comment. It is not surprising that the ANZDATA registry has significantly more patients with kidney failure secondary to non-FSGS than FSGS as FSGS is rare condition. To avoid any bias, the following covariates which could affect the outcomes were included in the analyses; age at initial KRT, gender, race, first KRT treatment, body mass index, smoking status, comorbidities and dialysis era or transplant era. This is described in page 6, line 134 to 136.**

Outcomes: Outcome 1: The authors have already defined the kidney transplantation as a censor event in line 108, but not sure why transplantation was considered as censored event in line 109. I am not sure about the meaning of statement “Transplantation was considered a censoring event or time-varying covariate”, especially the time varying covariate.

**RESPONSE: Thanks for the comments. Our full dataset includes all patients with kidney failure needing renal replacement therapy. For analysing the patient survival on dialysis (outcome 1), we censored all patients in whom kidney transplantation was their first renal replacement therapy and only included patients whose first RRT was dialysis. In patients on dialysis as their first renal replacement therapy, subsequent transplantation was considered a censoring event or time-varying covariate. This is described in page 5 line 107 to 110.**

Outcome 2: What is the reason for investigating the second outcome, which is "dialysis-independent recovery of kidney function"? the number of cases for this condition is minimal, I believe.

**RESPONSE: Thanks for the comments. We have deleted this outcome from out manuscript.**

Outcome 3: I am also uncertain about the inclusion of kidney transplantation as a third outcome in the study. It appears to diverge from the main aim and objectives of the research, which focus on the impact of FSGS on outcomes such as death and graft failure.

**RESPONSE: Thanks for the comments. We respectfully disagree with the reviewer. The likelihood of transplantation is a very important and clinically relevant outcome. We strongly feel that it should be reported.**

Outcome 4: The ANZDATA Registry records the primary renal disease at the start of the study. As such, I am uncertain how the recurrence of the primary disease was analysed using ANZDATA.

**RESPONSE: Recurrence of primary disease is entered in the ANZDATA registry by the individual transplant unit or the treating nephrologist.**

With regards to Outcome 5, I am unsure of the rationale behind using the commencement of dialysis as an endpoint. Typically, the standard measure for graft survival is from the first kidney transplant until the failure of the graft. The authors noted that death was treated as a competing event, and it is unclear why death was censored for outcomes 2-5.

**RESPONSE: We thank the reviewer for the comment. The rationale for using the commencement of dialysis as an endpoint is that the patient will only be started on dialysis if there is graft failure. Otherwise, there wouldn’t be any other reason for starting dialysis.**

Statistical analysis: The statistical analysis plan used in the study lacks clarity, as the authors have mixed up standard survival models with competing risk analysis. Additionally, there is a lack of detailed information on the use of time-varying covariates in the survival analysis.

**RESPONSE: We respectfully disagree with the reviewer. The statistical section of this manuscript was written, and the analysis was done by a qualified biostatistician (EM) along with a senior statistician (EP). We strongly feel that the statistical aspect of this manuscript is well described.**

Results: The manuscript contains numerous findings, and the authors have included irrelevant outcomes, resulting in an unnecessarily lengthy document.

**RESPONSE: We respectfully disagree with the reviewer. The findings of this study and the outcomes described are clinically relevant for clinicians and patients. The findings of this study give a better understanding of the outcomes of this patient group. Registry studies are key in providing long-term data in managing rare diseases such as FSGS, and we feel that our study, similar to other ANZDATA studies, describes long-term outcomes of patients with kidney failure.**

Timeframe: The ANZDATA 2018 data appears to be quite outdated. It is recommended that the authors use more recent ANZDATA 2022 data to obtain a more accurate depiction of the latest findings. This would enable them to present the actual outcomes of the recent data more effectively.

**RESPONSE: In this study, we have included patients who started RRT from 1963 to Dec 2018. However, the extracted data included follow-up until July 2021. We strongly feel that updating the data to 2022 will not make any difference in the findings of the study, especially as there hasn’t been any significant change in practice as far as management of patients with kidney failure or FSGS is concerned.**

Once again, we thank you for reviewing our manuscript.

Sincerely

Dr Bhadran Bose

On behalf of the authors
